# The Global Epidemiological Transition in Cardiovascular Diseases: Unrecognised Impact of Endemic Infections on Peripheral Artery Disease

**DOI:** 10.1007/s44197-022-00049-1

**Published:** 2022-07-16

**Authors:** Paul A. Agius, Julia C. Cutts, Peige Song, Igor Rudan, Diana Rudan, Victor Aboyans, Mary M. McDermott, Michael H. Criqui, F. Gerald R. Fowkes, Freya J. I. Fowkes

**Affiliations:** 1 Burnet Institute, Melbourne, VIC Australia; 2 Melbourne School of Population and Global Health, University of Melbourne, Parkville, VIC Australia; 3 Department of Epidemiology and Preventive Medicine, Monash University, Melbourne, VIC Australia; 4 Department of Infectious Diseases, at the Peter Doherty Institute for Infection and Immunity, University of Melbourne, Melbourne, VIC Australia; 5 Centre for Global Health, Usher Institute of Population Health Sciences and Informatics, University of Edinburgh, Edinburgh, UK; 6 Department of Social Medicine, School of Public Health, Zhejiang University School of Medicine, Hangzhou, China; 7 Department of Cardiology, University Hospital Dubrava, Zagreb, Croatia; 8 Department of Cardiology, Dupuytren University Hospital, and U1094 Inserm & IRD, Limoges, France; 9 Department of Medicine and Preventive Medicine, Feinberg School of Medicine, Northwestern University, Chicago, IL USA; 10 Department of Family Medicine and Public Health, University of California San Diego, La Jolla, CA USA

**Keywords:** Peripheral arterial disease, Malaria, Tuberculosis, HIV, Epidemiology

## Abstract

An epidemiological transition in the prevalence of peripheral artery disease (PAD) is taking place especially in low- and middle-income countries (LMICs) where an ageing population and adoption of western lifestyles are associated with an increase in PAD. We discuss the limited evidence which suggests that infection, potentially mediated by inflammation, may be a risk factor for PAD, and show by means of an ecological analysis that country-level prevalence of the major endemic infections of HIV, tuberculosis and malaria are associated with the prevalence of PAD. While further research is required, we propose that scientists and health authorities pay more attention to the interplay between communicable and non-communicable diseases, and we suggest that limiting the occurrence of endemic infections might have some effect on slowing the epidemiological transition in PAD.

## Introduction

Cardiovascular diseases (CVDs) caused by atherosclerosis in major arteries consist primarily of three conditions: coronary heart disease, cerebrovascular disease, and lower extremity peripheral artery disease (PAD). PAD has been traditionally considered a disease of high-income countries (HICs), but in recent years, a global epidemiological transition has occurred, similar to that for coronary heart disease and stroke^1^. Between 2000 and 2010, the number of cases of PAD increased by 13.1% in HICs but by a much larger 28.7% in low- and middle-income countries (LMICs). In 2010, more than two-thirds (69.7%) of PAD cases were in LMICs, the majority in Southeast Asia and the Western Pacific [1]. Since 2010, this increasing trend has persisted, so that by 2015, 234 million people globally were living with PAD [2].

This shifting epidemiology of PAD in LMICs may reflect the changing demographics of populations in these regions [3]. The risks of developing PAD increase sharply with age and the recent rise in LMICs is likely related to populations living longer, especially with chronic diseases such as diabetes, as well as increased survival after myocardial infarction and stroke. Furthermore, due mainly to adoption of unhealthy lifestyles, the classic risk factors for PAD in HICs, namely cigarette smoking, diabetes mellitus, hypertension and hypercholesterolemia, are also increasing in LMICs [1, 2]. Whether the presence of endemic infectious diseases in LMICs might influence the development of PAD has received little attention. Understanding the possible role of endemic infections might provide additional information on how best to slow the epidemiological transition in PAD.

## Do Endemic Infections Increase the Risk of PAD?

Infection is a possible contributor to the pathogenesis of atherosclerosis, including PAD [4]. In immuno-epidemiological studies, antibodies to a wide range of pathogens, for example, *Chlamydia pneumonia*, *Helicobacter pylori* and cytomegalovirus, have been associated with an increased risk of PAD [5]. Whether common endemic infections are associated with a population’s risk of acquiring PAD, particularly in LMICs, has not been studied. Globally, the commonest major endemic infections which affect large groups in a population and may have chronic effects are human immunodeficiency virus (HIV), tuberculosis, and malaria. Recently, the possible influence of the SARS-CoV-2 virus is of interest but, unless of prolonged duration, is unlikely to have a significant effect on the development over many years of chronic atherosclerotic disease.

An association between positive HIV status and incident PAD has been observed in clinical and epidemiological studies, mostly in HICs [6–8]. In a case control study, in the Netherlands of 540 HIV patients and 524 controls, PAD occurred in 0.6% of controls but in 2.6% of HIV patients (*p* < 0.01) [7], and the increased risk was independent of age and smoking. In a cohort of 91,953 US veterans, incident rates of PAD were higher among HIV + than uninfected veterans (11.9 v 9.9 per 1,000 person years) and were still 19% higher after risk factor adjustment. Comparable findings have been observed in China [8] and Burundi [9] and have likewise taken account of possible confounding variables such as cardiovascular risk factors and anti-retroviral therapy.

Tuberculosis may be involved in the pathogenesis of CVDs [10], but only one study has examined the association with PAD [11]. In this population-based cohort study in Taiwan of 14,350 tuberculosis patients and 28,700 non-tuberculosis subjects, the incidence of PAD over a 6-year follow-up was 5.4 vs 3.7 per 1000 patient years in the tuberculosis vs control cohorts. The hazard ratio adjusting for age, sex, co-morbidities and socio-economic status, was 3.9. Although the increased risk of PAD in tuberculosis patients was substantial, further studies are required. Nevertheless, pathogenic mechanisms occurring in tuberculosis and in infection more broadly, such as increased expression of pro-inflammatory cytokines and immune activation, could promote atherogenesis, making a causal association between tuberculosis and PAD plausible.

Malaria has not been studied as a possible risk factor for PAD. However, malaria may indirectly influence the pathogenesis of atherosclerosis. After repeated exposure to the malaria parasite, naturally acquired immunity can develop, resulting in chronic asymptomatic infection. This is associated with elevated inflammatory markers [12], such as C-reactive protein known to be associated with the risk of PAD [2]. Malaria is also a common cause of low birth weight which has been related to an increased risk of PAD in adulthood [13]. Recently, a causal link has been hypothesised between malaria and development of hypertension [14] known to be associated with the risk of PAD [2]. Thus, an increased prevalence of PAD might conceivably occur in malaria endemic areas.

## Ecological Analysis Provides Tantalising Evidence of the Possible Importance of Endemic Infection Levels

**Fig. 1:**
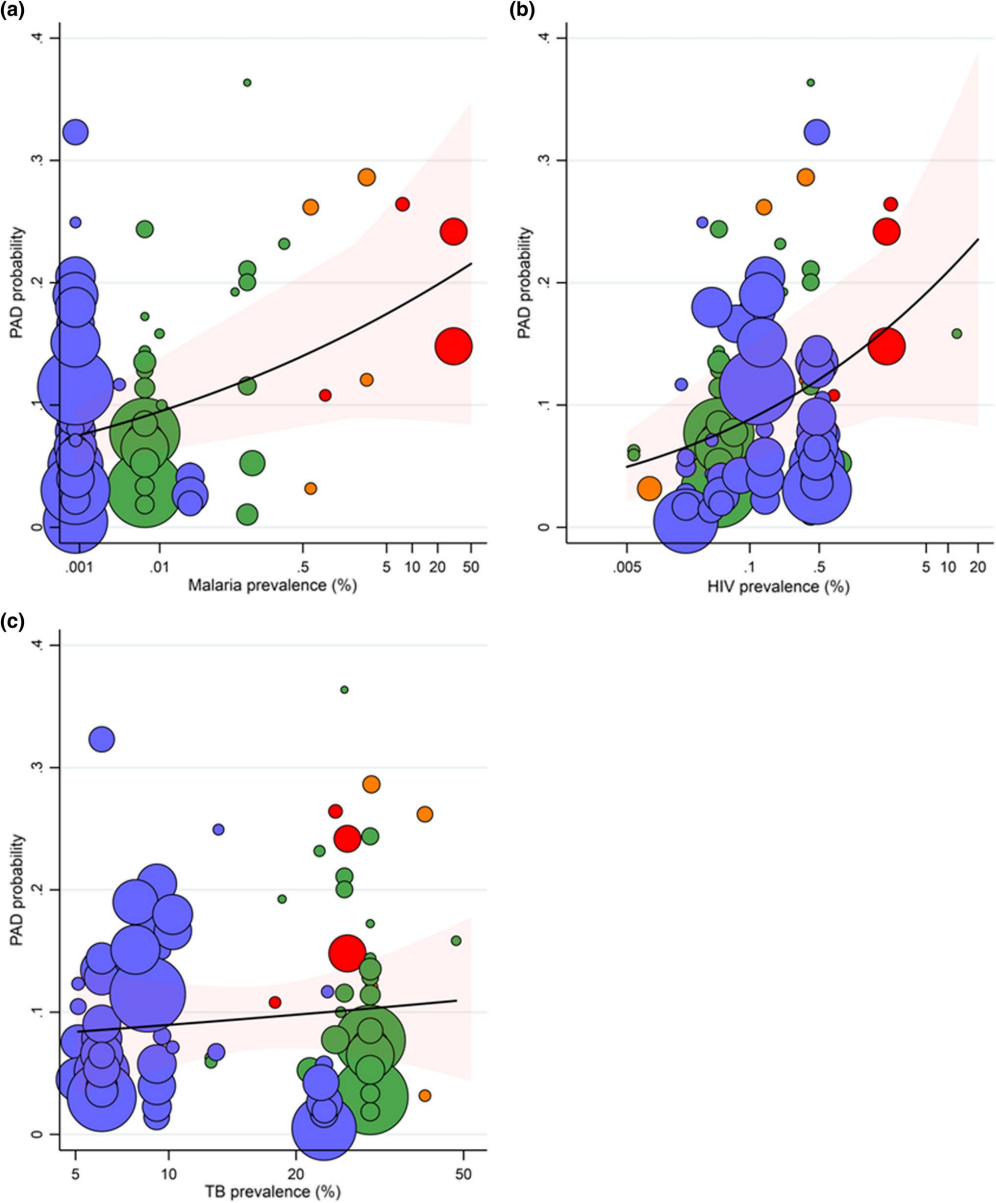
Prevalence of peripheral artery disease and country-level prevalence of malaria (**A**), HIV (**B**) and tuberculosis (**C**). *PAD* peripheral artery disease. *TB* tuberculosis. *HIV* human immunodeficiency virus. The black line shows average PAD probability (equivalent to PAD prevalence) by natural log prevalence of infection, with 95% confidence intervals shown by red shading. The circles depict individual studies by World Bank country income group [19] (red = low-income country, orange = low–middle-income country, green = upper middle-income country, blue = high-income country). Larger circles represent larger studies. The analysis used study-specific PAD cases (numerator) and respective sample sizes (denominator) to undertake binomial form logit estimation in generalised linear mixed modelling at two levels (level 1, studies; level 2, countries; and a random intercept for country)

Given the possibility that the occurrence of major infections might influence the development of PAD in endemic areas, we conducted an ecological analysis to determine whether prevalence of PAD was associated with rates of HIV, tuberculosis, and malaria. Applying generalised linear mixed modelling, we regressed study-specific PAD prevalence abstracted from 115 articles from 30 countries in our systematic review of global PAD in 2015 [2] on country-level HIV, tuberculosis and malaria prevalence estimates from the Global Burden of Disease study [15]. Figure 1 shows the results of individual studies of PAD probability (equivalent to prevalence) and infection prevalence. Overall, we found positive associations between prevalence of PAD and country-level prevalence of HIV (log odds = 0.22, *p* = 0.010), tuberculosis (log odds = 0.14, *p* = 0.619) and malaria (log odds = 0.11, *p* = 0.011), although the association between PAD and tuberculosis was not statistically significant. Quantifying these associations, a 20% relative increase in infection prevalence was estimated to yield approximately 4%, 3% and 2% increases in PAD prevalence for HIV, tuberculosis and malaria, respectively. This would be roughly equivalent, for example, to an increase in almost 10 million PAD cases globally associated with HIV in 2015.

Thus, this global ecological analysis has found an association between population frequencies of HIV, tuberculosis and malaria and prevalence of PAD. Ecological analyses should not be considered conclusive, particularly as there is no assessment of possible confounding factors. Nonetheless, our results, combined with those of studies demonstrating associations between infection and risk of PAD, as well as for other CVD outcomes, indicate a need for further research at a population level on the degree of co-morbidity of HIV, tuberculosis and malaria and PAD. An important issue to be addressed would be ethnicity. Africans, for example, have a particularly high risk of PAD unexplained by traditional cardiovascular risk factors and have high rates of HIV, tuberculosis and malaria [16]. Further work investigating plausible mechanisms on how these infections might increase the risk of PAD and subsequent prevalence of PAD in LMICs, is warranted.

## Need for Increasing Awareness of Interdependency Between Communicable and Non-communicable Disease

The possibility of the presence of endemic infection contributing to an increase in the frequency of PAD is one example of the need to consider the interdependency between communicable and non-communicable diseases. Traditionally, the prevention, control and treatment of communicable and non-communicable diseases have been approached separately at both national and local levels in HICs and LMICs. However, in recent years, the rapid increase in non-communicable diseases in LMICs, alongside the severe limitation in health care resources, has led to calls for greater integration in dealing with the two forms of disease [17]. Integration might include targeting public health interventions on populations experiencing dual morbidity. Encouragement might be given to incorporating non-communicable disease management into existing primary care systems in LMICs which tend to prioritise infection treatment and control. For example, providing simple inexpensive treatments to prevent major cardiovascular events and mortality in patients with PAD could be given priority [18]. Greater emphasis could be given to prevention overall including dealing with risk factors for both communicable and non-communicable diseases, such as smoking and diabetes mellitus, which increase the risk of both tuberculosis and PAD.

Although PAD is a common chronic atherosclerotic condition which causes loss of mobility and disability and is associated with reduced quality of life and an increased risk of major cardiovascular events and death, it is typically considered lower priority than acute cardiovascular conditions, such as myocardial infarction and stroke. However, in considering combined intervention strategies for HIV infection, tuberculosis and malaria and CVDs, the link between these infections and PAD should not be ignored and the rapid emergence of PAD in LMICs given the full attention it deserves.

## Conclusion

In this perspective piece, we have presented evidence to support the hypothesis that endemic infections may be a risk factor for PAD and demonstrated by means of an ecological analysis that country-level prevalence of common endemic infections are associated with the prevalence of PAD. Further research is required but also health authorities need to recognise that public health policies to limit endemic infections may have additional beneficial effects in slowing the epidemiological transition in chronic diseases such as PAD.

## Data Availability

Data available upon request.

## Abbreviations

CVDs: Cardiovascular diseases
HICs: High-income countries
LMIC: Low- and middle-income countries
PAD: Peripheral artery disease
